# N-Acetylcysteine for Polycystic Ovary Syndrome: A Systematic Review and Meta-Analysis of Randomized Controlled Clinical Trials

**DOI:** 10.1155/2015/817849

**Published:** 2015-01-08

**Authors:** Divyesh Thakker, Amit Raval, Isha Patel, Rama Walia

**Affiliations:** ^1^Department of Pharmacology, SAL Institute of Pharmacy, Ahmadabad, Gujarat 380060, India; ^2^Department of Pharmaceutical Systems and Policy, School of Pharmacy, West Virginia University, Morgantown, WV 26506, USA; ^3^Department of Biopharmaceutical Sciences, Bernard J. Dunn School of Pharmacy, Shenandoah University, Winchester, VA 22601, USA; ^4^Department of Endocrinology, Post-Graduate Medical Education and Research Institute (PGIMER), Chandigarh 160012, India

## Abstract

*Objective*. To review the benefits and harms of N-acetylcysteine (NAC) in women with polycystic ovary syndrome (PCOS). *Method*. Literature search was conducted using the bibliographic databases, MEDLINE (Ovid), CINAHL, EMBASE, Scopus, PsyInfo, and PROQUEST (from inception to September 2013) for the studies on women with PCOS receiving NAC. *Results*. Eight studies with a total of 910 women with PCOS were randomized to NAC or other treatments/placebo. There were high risk of selection, performance, and attrition bias in two studies and high risk of reporting bias in four studies. Women with NAC had higher odds of having a live birth, getting pregnant, and ovulation as compared to placebo. However, women with NAC were less likely to have pregnancy or ovulation as compared to metformin. There was no significant difference in rates of the miscarriage, menstrual regulation, acne, hirsutism, and adverse events, or change in body mass index, testosterone, and insulin levels with NAC as compared to placebo. *Conclusions*. NAC showed significant improvement in pregnancy and ovulation rate as compared to placebo. The findings need further confirmation in well-designed randomized controlled trials to examine clinical outcomes such as live birth rate in longer follow-up periods. Systematic review registration number is CRD42012001902.

## 1. Introduction 

Polycystic ovary syndrome (PCOS) is one of the most common endocrine disorders, affecting approximately 5% to 15% of women of reproductive age [1–3]. PCOS is mainly associated with anovulation, infertility, insulin resistance, and hyperandrogenism leading to metabolic disorders such as diabetes and cardiovascular diseases [4–6]. Treatment remains a challenge for women with PCOS. Although clomiphene citrate (CC) is the first-line of treatment for chronic anovulation among women with PCOS, failure to ovulate after receiving 150 mg/day is common and occurs in approximately 15% to 40% of women [7]. For those who do not respond to CC, there are very few therapies that can be tried before moving on to gonadotropin therapy or laparoscopic ovarian drilling (LOD). CC treatment has shown discrepancy between ovulation rates (75% to 80%) and conception rates (30% to 40%) unlike LOD treatment used in women with CC resistant PCOS [8]. The discrepancy might persist to a certain extent with gonadotropin treatment as well [9]. Insulin-sensitizing agents have been explored for treating the underlying cause of disorders associated with insulin resistance. Metformin, a widely used oral biguanide for treating type 2 diabetes, decreases the levels of insulin and androgens and increases the level of sex-hormone-binding globulin, thereby improving the endocrine parameters such as glucose tolerance and ovulation rates in women with PCOS [10]. However, a recent Cochrane review revealed that even though metformin was associated with improved clinical pregnancy and ovulation rate, it did not improve live birth rates when used alone or in combination with clomiphene or when compared with clomiphene [11]. Therefore, there is need for developing therapeutic options for treating the women with PCOS.

N-Acetyl cysteine (NAC) is a commonly used safe mucolytic drug, In addition, NAC increases the cellular levels of antioxidant and reduces glutathione at higher doses. Therefore, NAC has a potential to improve insulin receptor activity in human erythrocytes and improve insulin secretion in response to glucose [13]. Improvement in insulin receptor activity in hyperinsulinemic subjects can lead to a secondary decrease in the *β*-cell responsiveness to the oral glucose tolerance test. Decreased levels of circulating insulin can lead to significant reduction in Testosterone levels and free androgen index in women responding to the treatment [13, 14]. Advantages resulting from administration of NAC include prevention of endothelial damage resulting from oxidants in noninsulin-dependent adult diabetic subjects and biological effects such as, protection against focal ischemia, inhibition of phospholipid metabolism inhibition, proinflammatory cytokine release, and protease activity [14]. Therefore, it was suggested that the above effects exerted by NAC at the ovarian level may be as beneficial as its insulin-enhancing effects in inducing ovulation. In the absence of effective treatment options for PCOS, establishment of data on new options like NAC as monotherapy or supportive therapy may provide valuable information. There is no systematic review assessing effectiveness of NAC in PCOS. The present systematic review aims to assess the benefits and harms of NAC therapy in women with PCOS.

## 2. Objective

The purpose of this study was to determine if NAC therapy was more effective and safe in women with PCOS compared to placebo/metformin.

## 3. Materials and Methods 

### 3.1. Types of Studies, Interventions, Inclusion, and Exclusion Criteria

We included randomized studies in which NAC was compared to placebo or other agent(s) or NAC in combination with another drug to another class of drug alone. We excluded quasi- or pseudorandomized controlled trials or if the trails did not have a control group. In case of cross-over trials, we used data only from the first phase, that is, before cross-over of women with PCOS. Polycystic ovary syndrome had to be diagnosed according to the European Society for Human Reproduction and Embryology (ESHRE) and American Society for Reproductive Medicine (ASRM) sponsored PCOS Consensus Workshop criteria (the Rotterdam criteria) [15] or the National Institutes of Health (NIH) consensus criteria [16]. If the diagnostic criteria were not clearly stated in the trial, we contacted the trial authors for clarification. If clarification was not available, we excluded the trial.

The primary outcomes of this study were live birth rate per woman randomized and clinical pregnancy rate per woman randomized. Clinical pregnancy was defined as the presence of a gestational sac on ultrasound, as confirmed by the presence of a fetal heart rate or number of follicles produced per treatment cycle. Secondary outcomes were related to the safety. They included the following: ovarian hyperstimulation syndrome (OHSS) rate per woman randomized, defined according to the definition adopted by the reporting authors; miscarriage rate per woman randomized, where miscarriage was defined as the involuntary loss of a pregnancy before 20 weeks of gestation; and multiple pregnancy rate per woman randomized, where multiple pregnancy was defined as more than one intrauterine pregnancy. Other outcomes assessed in the study were resumption of menstrual regularity and spontaneous ovulation. Resumption of menstrual regularity was defined as initiation of menses or significant shortening of cycles. Number of women with resumption of normal menstrual cycle was defined as being between 21 and 34 days. Resumption of spontaneous ovulation was documented by biochemical methods, that is, measuring progesterone, where ovulation was defined as the evidence of serum progesterone in the luteal range of the reference laboratory, or a basal body temperature rise by >0.4°C for 10 days or more, as measured on a basal body temperature chart. Further, we also assessed other outcomes like improvement in body mass index (BMI), testosterone level, fasting glucose, fasting insulin, glucose/insulin ratio, and homeostatic model assessment-insulin resistance (HOMA-IR).

### 3.2. Search Strategy and Data Extraction

Two authors independently (DT, AR) ran electronic search strategy. It involved conducting a literature search for all pertinent published studies on the use of NAC for PCOS in terms of restoration of menstruation, induction of ovulation, and pregnancy using the bibliographic databases like the Cochrane Central Register of Controlled Trials (CENTRAL) in the Cochrane Library (from inception to September 2013), MEDLINE (Ovid) (from inception to September 2013), Scopus (from inception to September 2013), CINAHL (from inception to September 2013), and PsycINFO (from inception to September 2013). The references provided in selected articles identified were hand-searched to find additional studies. We also used ProQuest and ISI-Web of Science database for additional relevant citations. We contacted known experts and personal contacts regarding any unpublished materials.

Search terms included were: “Polycystic ovary syndrome” and “N-acetylcysteine” or “NAC” and “Hyperandrogaenemia”. The details of complete search strategies and results on number of hits are presented in Appendix 1 (in Supplementary Material available online at http://dx.doi.org/10.1155/2014/817849).

### 3.3. Data Extraction and Management

The PRISMA (preferred reporting items for systematic reviews and meta-analyses) flowchart was used for study selection [23]. Two authors independently appraised the methodological quality of the studies using the Cochrane Collaboration's tool for assessing risk of bias, a six-item quality assessment instrument. This tool evaluates the following areas: method of randomization, concealment of allocation, blinding, completeness of follow-up, selective outcome reporting, and other sources of bias [24]. As per the Cochrane Handbook for Systematic Reviews of Interventions [16], we stated any important concerns about bias that were not addressed in the other domains, that is, any baseline imbalance in factors strongly related to outcome measures. We rated the studies as “high,” “low,” or “unclear” risk of bias in each domain. An “unclear” judgment was made if insufficient detail on what happened in the study was reported; if what happened in the study was known but the risk of bias is unknown; or if an entry was not relevant to the study at hand (particularly for assessing blinding and incomplete outcome data, when the outcome being assessed had not been measured in the study report). Two authors (AR, DT) independently entered data into a data extraction form about the study characteristics including methods, participants, interventions, and outcomes. Any disagreement was resolved by referring to the trial report and through discussion and consultation with a third author (RW). If data from the trial reports were insufficient or missing, we contacted the investigators of the studies for additional information. Where possible, we extracted data to allow an intention-to-treat analysis. If the number randomized and the analyzed were inconsistent, we calculated the percentage loss to follow-up and reported this information in an additional table.

### 3.4. Data Analysis

We calculated a summary statistic for each outcome with respect to the interventions using a fixed-effect model in RevMan 5.2 software. We used the Peto odds ratio (OR) as a measure of effect for each dichotomous outcome and the mean difference (MD) for each continuous outcome. If the data were reported using geometric means, we extracted standard deviations on the log scale. We contacted study authors for missing data. If missing data were not available from the authors, we did not use the data in the analysis. Heterogeneity was assessed using Chi-square statistic. A low *P* value or large Chi-sqaure statistic relative to the degree of freedom indicates heterogeneity. The *I* ^2^ statistic was used to quantify the heterogeneity [25]. Subgroup analyses were performed based on type of comparison, duration of intervention, and ethnicity of participants, to investigate source of heterogeneity. Publication bias was assessed using funnel plot. We also performed sensitivity analyses to examine the impact on the results in relation to a number of factors including comedication, quality of allocation concealment, blinding, intention-to-treat analysis, source of funding, and different diagnostic criteria of PCOS or obesity [26].

## 4. Results 

**Figure 1:**
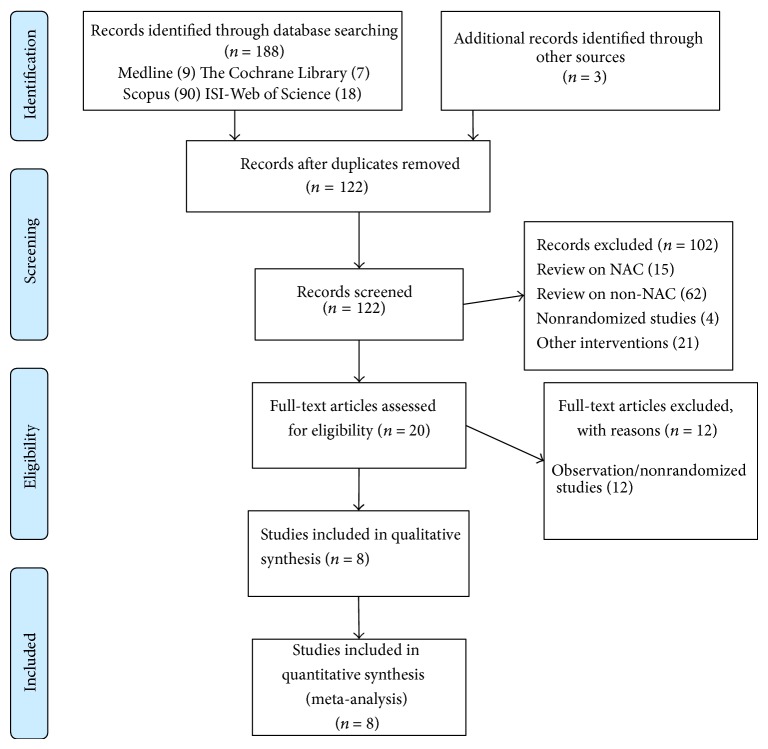
PRISMA flow diagram for selection of studies for the systematic review. Flow diagram style adapted from Moher et al. [12].

We retrieved 191 articles (MEDLINE: 9, The Cochrane library: 7, PsycINFO: 0, Scopus: 90; CINAHL: 26; ISO-Web of Science: 18; ProQuest: 38, and 3 citations using conference proceedings and hand-searching of journals). Two studies were excluded due to lack of a control group and had a prepost study design for evaluating treatment [13, 27]. After excluding narrative reviews, nonrandomized studies, intervention studies on agents other than NAC, and duplicate publications, we included eight studies (eight articles) [14, 17–22, 28].  Figure 1 describes the selection procedures for eight studies using the PRISMA flow diagram.

**Figure 2:**
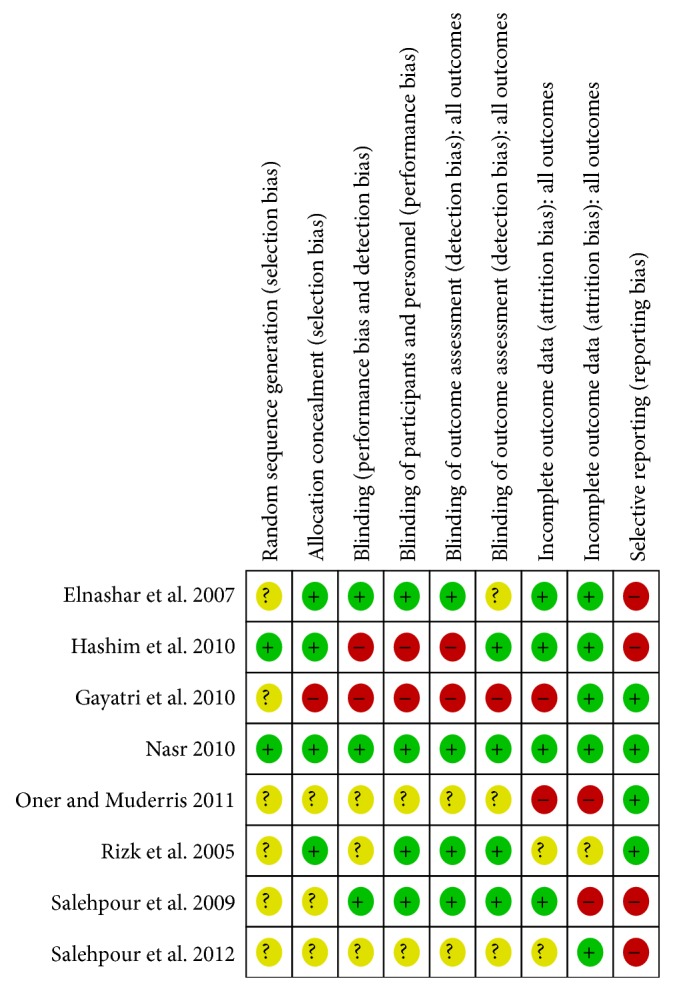
Cochrane risk of bias rool summary for included studies.

Figure 2 describes the summary of risk of bias among the included studies. The methods for randomization were unclear in six studies. Only two studies described the use of computer generated randomization list for sequence allocation [21, 22]. Treatment allocation was concealed by administration of third party (nurse) using opaque sealed envelopes in four studies [14, 20–22], unclear in three studies [18, 19], and not done in one study [28]. Only four studies had low-risk of performance bias due to proper blinding [14, 17, 20, 22], and two studies [21, 28] were open-label with high risk of performance bias, while in remaining two studies blinding was unclear. Four studies [14, 17, 19, 20] had high risk of selective reporting bias especially on primary outcomes and safety outcomes. Three studies reported outcomes, which were not specified in the protocol. Those outcomes were homeostasis model assessment for insulin resistance (HOMA-IR) and Ferriman-Gallwey scale, fasting glucose, fasting insulin and glucose/insulin ratio, lipid profile and TNF-alpha, acne, infertility, and weight gain and testosterone level. Two studies had high risk of attrition bias with attrition rate of more than 20% [17, 18].

Characteristics of included studies are provided in Table 1. All the studies except Rizk et al. provided the diagnostic criteria, modified Rotterdam criteria, for PCOS. In Rizk et al., PCOS was diagnosed only by a finding of bilaterally normal or enlarged ovaries (ovarian volume < 12 cm^3^) with the presence of at least 7 to 10 peripheral cysts per ovary [20]. All the studies confirmed absence of the following diseases: hyperprolactinaemia, Cushing's disease, and androgen-secreting tumors. Three studies included women with PCOS (*n* = 261) as main inclusion criteria, while five studies included women with clomiphene-resistant PCOS (*n* = 649) as main inclusion criteria. Clomiphene resistance was identified as 100 mg CC daily for 5 days per cycle for at least three cycles for persistent anovulation, in one study and 150 mg CC daily for 5 days per cycle for at least three cycles for persistent anovulation, in other three studies. All the studies included women with only reproductive age ranging from 18 to 39 years. Presence of diabetes, thyroid disorders, and use of medications affecting glucose metabolism were main exclusion criteria in all the included studies. One study had used treatment following laparoscopy.

Eight studies with a total of 910 women with PCOS were randomized to NAC and placebo or metformin. Four studies randomized 441 women to NAC and placebo in 1 : 1 randomization ratio, while remaining four randomized 469 women to NAC or metformin in 1 : 1 randomization ratio. All the included studies were published in English and carried out at single academic medical center associated with university in Middle East (Egypt, Iran, and Turkey) and one in Asia (India). The number of women participating in each trial varied from 60 to 192 with an average of 113 women per study. The study duration ranged from 2 to 12 months. Overall, a total of 842 women completed the studies with overall attrition rate of 9.3% in all the studies.

In studies of women with PCOS, two studies reported concurrent use of clomiphene citrate. All the studies asked to use normal diet and maintain normal lifestyle habit during studies. Baseline characteristics of included studies are shown in Table 2. The mean age of included studies ranged from 20 years to 33 years. One study had obese women (BMI > 30 kg/m^2^) [20], six studies had moderately obese women (BMI: 25–30 kg/m^2^) [14, 17–19, 21, 22], and one study had nonobese women (BMI: 20–25 kg/m^2^) [28]. Only four studies reported rate of menstrual irregularity and amenorrhea or oligomenorrhea, which ranged from 6% [21] to 17% [22] and from 23% [14] to 93.7% [21], respectively. Three studies reported hirsutism with prevalence ranging from 4% to 61.1% [17, 22, 28] while two studies reported problems with acne, ranging from 2% to 27.8% [17, 28]. Six studies reported duration of infertility, of which the four studies reported mean duration of infertility around 4 to 5 years [14, 17, 19, 20] while for one study it was as high as 10 years of infertility [28]. Women had normal fasting glucose level in all the included studies.

**Figure 3:**
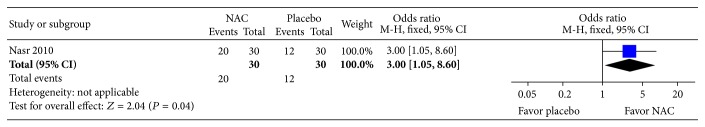
Forest plot: outcome: live birth rate in women with PCOS comparing NAC with placebo.

**Figure 4:**
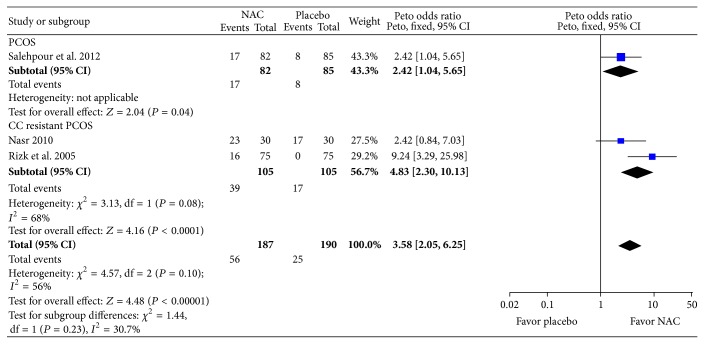
Forest plot: outcome: pregnancy rate in women with PCOS comparing NAC with placebo.

**Figure 5:**
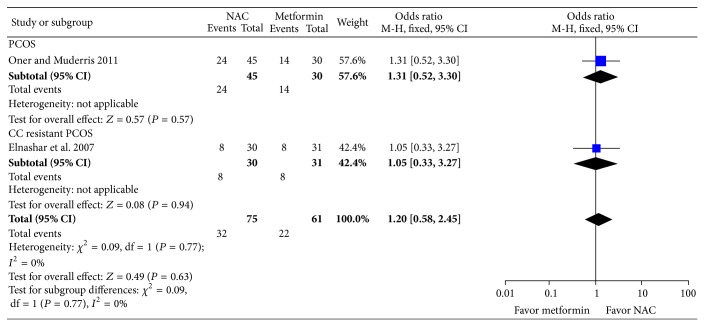
Forest plot: outcome: pregnancy rate in women with PCOS comparing NAC with metformin.

Primary outcome of this review was live-birth rate which was assessed in a single study [22]. The odds of live birth with NAC was nearly three times higher in women with PCOS as compared to placebo (Peto odds ratio, pOR: 3.00; 95% CI: 1.05, 8.60; *P* = 0.04; 1 trial; 60 women) (Figure 3). Five studies assessed pregnancy rate per woman. Compared to placebo, women with NAC were around three and half times more likely to have pregnancy (pOR: 3.58; 95% CI: 2.05, 6.25; *P* < 0.0001; *I* ^2^ = 56%; 3 trials; 377 women) (see Figure 4). In subgroup based on PCOS status, CC resistant PCOS, or PCOS, it was found that, in women with CC resistant PCOS, NAC increased likelihood, around five times, of pregnancy compared to placebo (pOR: 4.83; 95% CI: 2.30–10.13; *P* < 0.0001; *I* ^2^ = 68%; 2 trials; 210 women). In contrast, compared to metformin, women on NAC were 60% less likely to have pregnancy (pOR: 0.40; 95% CI: 0.23, 0.71; *I* ^2^ = 70; 2 trials; 290 women) without considerable heterogeneity among the studies (see Figure 5). Only three studies reported normal semen analysis of partner while assessing pregnancy outcomes.

**Figure 6:**
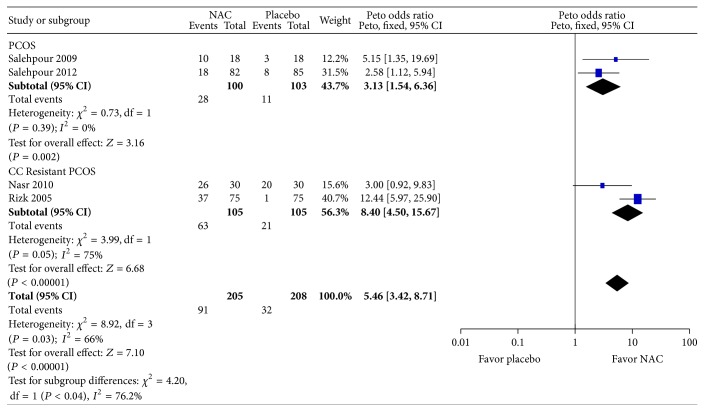
Forest plot: outcome: ovulation rate in women with PCOS comparing NAC with placebo.

**Figure 7:**
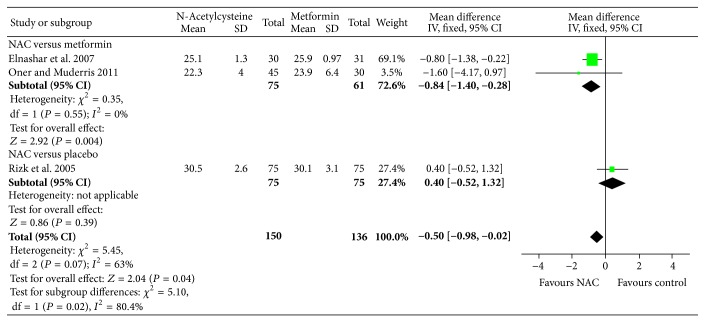
Forest plot: outcome: body-mass index (BMI) (kg/m^2^) in women with PCOS comparing NAC with placebo/metformin.

**Figure 8:**
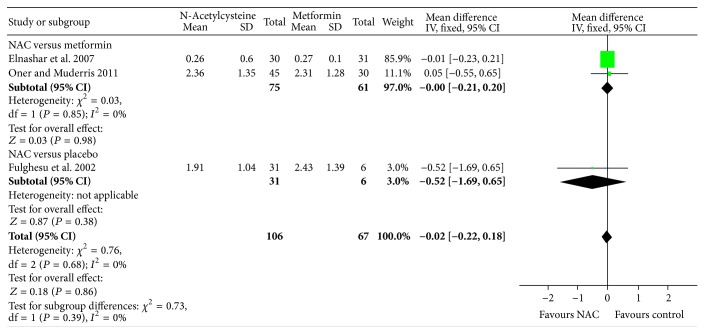
Forest plot: outcome: testosterone level (nmol/L) in women with PCOS comparing NAC with placebo/metformin.

**Figure 9:**
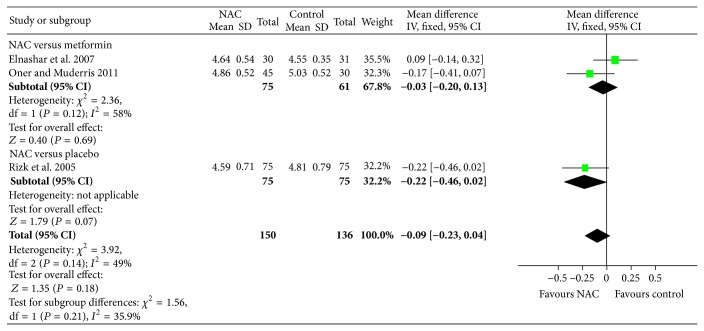
Forest plot: outcome: fasting glucose (mg/dL) in women with PCOS comparing NAC with placebo/metformin.

In terms of secondary outcomes, there was no significant difference in the miscarriage per woman compared to placebo (Peto OR: 1.28; 95% CI: 0.35, 4.70; *P* = 0.71; *I* ^2^ = 82%; 2 trials, 190 women) with considerable heterogeneity. Six studies reported ovulation rate. Compared to placebo, women on NAC were three times as likely to have ovulation (pOR: 3.13; 95% CI: 1.54, 6.36; *P* = 0.002; *I* ^2^ = 0%; 2 trials, 200 women) in women PCOS, and, for CC-resistant PCOS, women on NAC were nine times as likely to have ovulation (pOR: 8.40; 95% CI: 4.50, 15.67; *P* = 0.04; *I* ^2^ = 77.5%; 2 trials, 210 women) with considerable heterogeneity (see Figure 6). However, this association was in opposite direction for the comparison between NAC and metformin. Compared to metformin, women on NAC were 87% less likely to have ovulation (pOR: 0.13; 95% CI: 0.08, 0.22; *P* < 0.001; *I* ^2^ = 0; 2 trials, 253 women). All the studies reported mild adverse events, however, did not describe the nature of adverse event in details. Two studies reported no incidence of OHSS in any of the groups. No cases of OHSS were reported. There was no significant difference in rate of menstrual regulation with NAC compared to placebo (pOR: 3.00; 95% CI: 0.92, 9.83; *P* = 0.07; 1 trial; 60 women) or metformin (Peto OR: 1.20; 95% CI: 0.58, 2.45; *P* = 0.63; *I* ^2^ = 0%; 2 trials, 136 women). Due to difference in the reporting and measurement of acne and hirsutism across the studies, meta-analysis was not feasible; however studies showed that there was no difference in acne/acne severity, hirsutism rate/severity of hirsutism following NAC treatment compared to placebo, or metformin. Further, compared to metformin, NAC significantly reduced BMI (MD: −1.00; 95% CI: −1.49, −0.52; *P* < 0.0001; *I* ^2^ = 0%, 3 studies; 236 women), while there was no significant change in BMI following NAC treatment compared to placebo (see Figure 7). Addressing the hyperandrogenism, NAC reduced total testosterone level (MD: −0.19; 95% CI: −0.29, −0.10; *P* = 0.0001; *I* ^2^ = 0%; 2 trials; 175 women) compared to metformin but did not show any difference in testosterone level (MD: −0.83; 95% CI: −1.79, 0.13; *P* = 0.09; 1 trial, 36 women) compared to placebo (see Figure 8). Compared to metformin or placebo, NAC significantly reduced fasting glucose levels in women with PCOS (see Figure 9). In addition, compared to placebo or metformin, NAC did not significantly improve fasting insulin or HOMA-IR.

## 5. Discussion 

This review was conducted to assess clinical benefits and harms of NAC among women with PCOS. A total of eight randomized controlled trials with 910 women compared effects of NAC with placebo or metformin in women with PCOS. NAC significantly improved rates of live births and spontaneous ovulation compared to placebo in women with PCOS. However, we found no evidence of effects of NAC on improving menstrual regularity, acne, hirsutism, BMI, fasting insulin, fasting glucose, or HOMA-IR. NAC was not associated with greater benefits to metformin for improving pregnancy rate, spontaneous ovulations, and menstrual regularity. Metformin also improved the BMI, total testosterone, insulin level, and lipid levels compared to NAC. The side effect profiles were mild to moderate with no serious adverse drug events reported. Minor side effects were not reported in detail. All the studies were of short duration (three months) and long-term data on the comparative effects of NAC are lacking for important clinical outcomes such as resumption of menstrual regularity.

We aimed to minimize the risk of bias to provide good quality of systematic review. Therefore, we included only randomized controlled clinical trials, ideally with proper randomization, allocation concealment, blinding, and free from selective reporting. However, not all the studies fulfilled all these criteria. The methods used for randomization, allocation concealment, and blinding were not clearly reported by seven out of eight studies. None of the studies has adjusted for baseline difference in characteristics. Two studies had attrition rate of more than 20% [17, 18]. Four studies did not report testing on semen quality of partner which would be a critical factor for pregnancy rates and live birth rates [14, 17, 18, 28]. The studies assessing the pregnancy rate should assess the semen analysis of the partner. Only three studies assessed for normal semen analysis [14, 17, 18]. Five studies did not mention the restriction on the use of body hair removal methods while assessing hirsutism as an outcome. In addition, there is a need to have proper blinding of participants and investigators to prevent observation biases while assessing subjective outcomes such as hirsutism and acne [17, 18, 20, 22, 28]. Theoretically, studies of a relatively short duration could demonstrate a significant impact on clinical outcomes such as menstrual regularity or ovulation rate, although this is somewhat unlikely, even with regards to important adverse events. Only one study evaluated the long-term efficacy and safety of NAC in women with PCOS and live birth rate as an outcome [22].

There are no other systematic reviews on NAC for women with PCOS. This review is the first to generate the hypothesis on the use of NAC for PCOS. In order to limit bias in the review process, the search strategies exclusively performed to get both formal and nonformal sources of information without any restrictions on language of the search. The study selection, risk of bias assessment, and data collection were conducted independently by two review authors but without blinding. Any disagreement was resolved by discussion with the third review author. We did not exclude any study due to lack of additional information. Failure to obtain the primary study data in analyzable format was the main limitation of the review. It was not feasible to perform sensitivity analyses to determine whether there was an effect on outcome from allocation concealment, blinding, or obesity due to insufficient data. In addition due to limited studies, we were not able to see publication bias. A number of the results were constrained by small numbers of participants and wide confidence intervals, which limited the precision and confidence of the conclusions. Meta-analysis was not possible for a number of primary and secondary outcomes, comparing NAC to placebo due to either an absence of trials or the presence of a single trial only. In the future, well-designed RCTs with large sample sizes are warranted to confirm or refute the current evidence.

## 6. Conclusion 

NAC showed significant improvement in pregnancy and ovulation rate in the studies with short-term outcomes compared to placebo. However, the given the limitations of existing studies such as poor quality, less studies assessing live-birth rates, in future, well-designed randomized-controlled trials should conducted.

## Supplementary Material

Appendix of the current paper “Thakker D, Raval A, Patel I, Walia R. N-Acetylcysteine for Polycystic Ovary Syndrome: A Systematic review and Meta-analysis of Randomized Controlled Clinical Trials” includes the details on electronic search strategies conducted using Medline (Ovid), The Cochrane Library, Scopus, CINAHL and PsycInfo (EBSCOhost).

## Figures and Tables

**Table 1:** Characteristics of included studies: study information, treatments, inclusion and exclusion criteria systematic reviews of randomized controlled trials.

Study ID	Study period	Country	Treatment arms	Study duration	Diagnosis criteria	Inclusion criteria	Exclusion criteria
Among women with polycystic ovary syndrome							
Salehpour et al. 2009 [17]	Feb 2007–February 2008	Iran	NAC: 1800 mg/day, divided into three daily doses; placebo: ORS, divided into three daily doses	6 months	Rotterdam criteria, ESHRE/ASRM 2003	Presence of PCOS; spontaneous onset of maturation; and normal sexual development	Diabetes mellitus; use of medications affecting glucose metabolism Use hormonal analogues other than progesterone Severe hepatic or kidney diseases; active peptic ulcer
Gayatri et al. 2010 [28]	June 2006–December 2007	India	NAC: 1800 mg/day, orally divided in three doses; metformin: 500 mg/day for week 1; 500 mg twice daily for week 2 and 500 mg thrice daily afterwards	3 months	Rotterdam criteria, ESHRE/ASRM 2003	Presence of PCOS	Diabetes mellitus; use of medications affecting glucose metabolism Use hormonal analogues other than progesterone Severe hepatic or kidney diseases; active peptic ulcer
Oner and Muderris 2011 [18]	March 2008–April 2009	Turkey	NAC: 1800 mg/day, orally divided in three doses; metformin: 1500 mg/day, orally divided in three doses	6 months	Rotterdam criteria, ESHRE/ASRM 2004	Presence of PCOS	Diabetes mellitus; thyroid disease Use of any drugs that could interfere with the normal function of the hypothalamic-pituitary-gonadal axis
Salehpour et al. 2012 [19]	Jan 2008–Dec 2009	Iran	NAC: 1200 mg/day, divided into two daily doses; Placebo: ORS, divided into two daily doses	3 months	Rotterdam criteria, ESHRE/ASRM 2004	Presence of PCOS; Age 20–35 years; Infertility duration less than 10 years; BMI <35 kg/m^2^; Normal semen analysis	Thyroid dysfunction; History of large ovarian cyst formation (>6 cm); History of visual disturbance caused by CC; History of asthma and or allergy to medications; Use of medications affecting glucose metabolism; Use hormonal analogues other than progesterone;

Among women with clomiphene resistant polycystic ovary syndrome							
Rizk et al. 2005 [20]	March 2002–Nov 2003	Egypt	NAC:		Other	Presence of CC resistant PCOS; Age 18–39 years	Thyroid disfunction; Allergy to medications; Use of medications affecting glucose metabolism; Use hormonal analogues other than progesterone;
Elnashar et al. 2007 [14]	Dec 2004–Dec 2005	Egypt	NAC: 1800 mg/day, orally divided in three doses; Metformin: 1500 mg/day, orally divided in three doses	2 months	Rotterdam criteria, ESHRE/ASRM 2003	Presence of CC resistant PCOS; Age 18–39 years; Period of infertility >2 years	History of pelvic surgery or infertility factor other than anovulation; Patients with hyperglycemia (fasting blood sugar of <100 mg/dL)
Hashim et al. 2010 [21]	Jan 2005–June 2009	Egypt	NAC: 1800 mg/day, orally divided in three doses; Metformin: 1500 mg/day, orally divided in three doses	3 Months	Rotterdam criteria, ESHRE/ASRM 2003	Presence of CC resistant PCOS	Diabetes mellitus; Use of medications affecting glucose metabolism; Use hormonal analogues other than progesterone; Smoking & alcohol use; Age more than 40 years
Nasr 2010 [22]	April 2007–June 2009	Egypt	NAC: 1200 mg/day, divided into two daily doses; Placebo: ORS, divided into two daily doses	12 months	Rotterdam criteria, ESHRE/ASRM 2003	Presence of CC resistant PCOS; Age 18–38 years; >2 years with infertility; Patent fallopian tubes & Normal semen analysis	Use hormonal analogues other than progesterone; contraindications to laparoscopy or general anaesthesia

Note: All the studies were carried out in single center within academic medical centers. Rotterdam European Society for Human Reproduction and Embryology/American Society for Reproductive Medicine-sponsored PCOS Consensus Workshop, that is, the presence of at least two of the following three criteria: (1) oligo- or anovulation, (2) clinical and/or chemical signs of hyperandrogenism, and/or (3) polycystic ovaries; and exclusion of other aetiologies such as congenital adrenal hyperplasia, Cushing's syndrome or androgen-secreting tumours. Clomiphene Citrate (CC) resistant was defined as 100 mg CC daily for 5 days per cycle for at least three cycles for persistent anovulation in Rizk et al. 2005 [20] and 150 mg CC daily for 5 days per cycle for at least three cycles for persistent anovulation in other studies. Gayatri et al. 2010 [28] used 50 mg/day of CC from day 2 to 6 and gradual increment in next cycle by 50 mg/day with maximum up to 150 mg/day. None of the included studies were funded by commercial funding agencies like Pharmaceutical Industries. However, drugs for the studies were provided by the Pharmaceutical Companies. All the women were asked to have normal life-style and eating habit during the study.

**Table 2:** Characteristics of included studies: baseline characteristics systematic reviews of randomized controlled trials.

Characteristics	Among women with PCOS			Among women with clomiphene resistant PCOS				
	Salehpour et al. 2009 [17]	Gayatri et al. 2010 [28]	Oner and Muderris 2011 [18]	Salehpour et al. 2012 [19]	Rizk et al. 2005 [20]	Elnashar et al. 2007 [14]	Hashim et al. 2010 [21]	Nasr 2010 [22]
	NAC/placebo	NAC/metformin	NAC/metformin	NAC/placebo	NAC/placebo	NAC/metformin	NAC/metformin	NAC/placebo
Total randomized, *n*	46	115	100	180	153	64	192	60
Randomised, *n*	46	56/59	50 /50	90/90	NA	32/32	95/97	30/30
Completed, n	36	50/50	45/31	82/85	75/75	30/31	97/95	30/30
Total, n	36	100	76	167	150	61	192	60
Attrition rate	21.7%	13%	24%	7%	2%	4.7%	0%	0%
*Baseline Characteristics *								
Age, years	27.2 (5.4) 27.8 (6.1)	22.6 (3.8); 23.2 (4.1)	23.7 (4.4); 22.6 (4.8)	27.22 (3.32); 27.41 (3.41)	28.9 (4.7); 28.4 (5.7)	27.33 (3.35); 26.73 (5.36)	27.3 (2.6); 26.8 (2.2)	28.4 (4.2); 29.2 (3.7)
Weight, kg	74.1 (11.7) 74.1 (13.2)	70.5 (3.45); 69.8 (8.32)	NA	NA	101.3 (12.4); 99.2 (12.3)	NA	NA	NA
BMI, kg/m^2^	29.5 (4.1) 29.5 (4.4)	26.54 (2.35); 27.28 (3.25)	23 (4.6); 24.3 (6.2)	26.78 (2.24); 26.67 (2.01)	30.5 (2.6); 30.1 (3.1)	25.8 (0.94); 26.8 (1.52)	26.6 (2.2); 26.3 (2.3)	28.6 (3.7); 29.1 (4.2)
Amenorrhea patients (%)	2 (11.1%) 2 (11.1%)	4 (8%); 4 (8%)	NA	NA	NA	NA	6 (6.3%); 7 (7.2%)	5 (17%); 6 (20%)
Oligomenorrhea, *n* (%)	NA	29 (58%); 30 (60%)	NA	NA	NA	76.7 (23); 77.4 (24)	89 (93.7%); 90 (92.8%)	NA
Hirsutism, *n* (%)	11 (61.1%) 10 (55.6%)	2 (4%); 3 (6%)	NA	NA	NA	NA	NA	16 (53%); 18 (60%)
Acne, *n* (%)	5 (27.8%) 5 (27.8%)	1 (2); 2 (4)	NA	NA	NA	NA	NA	NA
Duration of Infertility, mean (SD)	4.5 (2.2) 4.2 (3.3)	10 (20); 8 (16)	NA	4.39 (1.96); 4.45 (1.94)	5 (2.9); 4.4 (2.6)	NA	4.5 (1.2); 4.7 (1.3)	5.3 (1.9); 4.9 (2.1)
Testosterone level, nmol/dL, mean (SD)	0.91 (0.48) 1.01 (0.45)	1.55 (0.29); 1.65 (0.24)	80.8 (41.1); 86.1 (48.4)	NA	NA	98.27 (31.5); 106.5 (44.6)	1.06 (0.27); 1.03 (0.31)	NA
FPG, mg/dL, mean (SD)	95.6 (10.9) 96.8 (27.7)	88.53 (5.14); 87.65 (4.34)	88.5 (6.8); 88.9 (7.4)	NA	81.9 (12.6); 85.9 (14.1)	83.3 (8.8); 85.9 (9.4)	91.3 (1.5); 89.6 (1.4)	NA
HOMA-IR	5.22 (5.58); 4.9 (4.2)	5.52 (1.35); 5.42 (1.36)	4.5 (1.2); 4.3 (0.9)	NA	NA	NA	NA	NA
