# Supplementary material for: N-Acetylcysteine for Polycystic Ovary Syndrome: A Systematic Review and Meta-Analysis of Randomized Controlled Clinical Trials
Source: Obstet Gynecol Int. 2015 Jan 8;2015:817849. doi: 10.1155/2015/817849 (PMC4306416; doi:10.1155/2015/817849)
Supplement: Supplementary file 1 — Appendix of the current paper “Thakker D, Raval A, Patel I, Walia R. N-Acetylcysteine for Polycystic Ovary Syndrome: A Systematic review and Meta-analysis of Randomized Controlled Clinical Trials” includes the details on electronic search strategies conducted using Medline (Ovid), The Cochrane Library, Scopus, CINAHL and PsycInfo (EBSCOhost). [file 817849.f1.docx]

**Appendix 1. Cochrane Central Register of Controlled Trials (CENTRAL) search strategy**

1 exp Polycystic Ovary Syndrome/
2 Polycystic Ovar$.tw.
3 PCO$.tw.
4 (stein-leventhal or leventhal).tw.
5 (ovar$ adj1 sclerocystic).tw.
6 (ovar$ adj1 degeneration).tw.
7 or/1-6
8 exp hydroxymethylglutaryl-coa reductase inhibitors/ or exp lovastatin/ or exp meglutol/ or exp pravastatin/ or exp simvastatin/
9 HMG coenzyme reductase.tw.
10 statin$.tw.
11 (Atorvastatin or Simvastatin).tw.
12 (Rosuvastatin or Lovastatin).tw.
13 (Mevastatin or Pravastatin).tw.
14 mevinolin.tw.
15 HMG-coA reductase$.tw.
16 HMG coenzyme A reductase.tw.
17 Zocor.tw.
18 meglutol.tw.
19 Hydroxymethylglutaryl CoA Reductase.tw.
20 Hydroxy 3 methylglutaryl CoA Reductase.tw.
21 CoA Reductase, 3-Hydroxy-3-methylglutaryl.tw.
22 Reductase, 3-Hydroxy-3-methylglutaryl CoA.tw.
23 or/8-22
24 7 and 23

**Appendix 2. MEDLINE**

1 exp Polycystic Ovary Syndrome/
2 Polycystic Ovar$.tw.
3 PCO$.tw.
4 (stein-leventhal or leventhal).tw.
5 (ovar$ adj1 sclerocystic).tw.
6 (ovar$ adj1 degeneration).tw.
7 or/1-6
8 exp hydroxymethylglutaryl-coa reductase inhibitors/ or exp lovastatin/ or exp meglutol/ or exp pravastatin/ or exp simvastatin/
9 HMG coenzyme reductase.tw.
10 statin$.tw.
11 (Atorvastatin or Simvastatin).tw.
12 (Rosuvastatin or Lovastatin).tw.
13 (Mevastatin or Pravastatin).tw.
14 mevinolin.tw.
15 HMG-coA reductase$.tw.
16 HMG coenzyme A reductase.tw.
17 Zocor.tw.
18 meglutol.tw.
19 Hydroxymethylglutaryl CoA Reductase.tw.
20 Hydroxy 3 methylglutaryl CoA Reductase.tw.
21 CoA Reductase, 3-Hydroxy-3-methylglutaryl.tw.
22 Reductase, 3-Hydroxy-3-methylglutaryl CoA.tw.
23 or/8-22
24 7 and 23
25 randomized controlled trial.pt.
26 controlled clinical trial.pt.
27 randomized.ab.
28 placebo.tw.
29 clinical trials as topic.sh.
30 randomly.ab.
31 trial.ti.
32 (crossover or cross-over or cross over).tw.
33 or/25-32
34 (animals not (humans and animals)).sh.
35 33 not 34
36 24 and 35

**Appendix 3. EMBASE**

1 exp ovary polycystic disease/
2 Polycystic Ovar$.tw.
3 PCO$.tw.
4 (stein-leventhal or leventhal).tw.
5 (ovar$ adj1 sclerocystic).tw.
6 (ovar$ adj1 degeneration).tw.
7 exp hydroxymethylglutaryl coenzyme A reductase inhibitor/
8 HMG coenzyme reductase.tw.
9 hydroxymethylglutaryl-coa reductase inhibitor$.tw.
10 statin$.tw.
11 (Atorvastatin or Simvastatin).tw.
12 (Rosuvastatin or Lovastatin).tw.
13 (Mevastatin or Pravastatin).tw.
14 mevinolin.tw.
15 HMG-coA reductase$.tw.
16 HMG coenzyme A reductase.tw.
17 Zocor.tw.
18 meglutol.tw.
19 Hydroxymethylglutaryl CoA Reductase.tw.
20 Hydroxy 3 methylglutaryl CoA Reductase.tw.
21 CoA Reductase, 3-Hydroxy-3-methylglutaryl.tw.
22 Reductase, 3-Hydroxy-3-methylglutaryl CoA.tw.
23 or/1-6
24 or/7-22
25 23 and 24
26 Clinical Trial/
27 Randomized Controlled Trial/
28 exp randomization/
29 Single Blind Procedure/
30 Double Blind Procedure/
31 Crossover Procedure/
32 Placebo/
33 Randomi?ed controlled trial$.tw.
34 Rct.tw.
35 random allocation.tw.
36 randomly allocated.tw.
37 allocated randomly.tw.
38 (allocated adj2 random).tw.
39 Single blind$.tw.
40 Double blind$.tw.
41 ((treble or triple) adj blind$).tw.
42 placebo$.tw.
43 prospective study/
44 or/26-43
45 case study/
46 case report.tw.
47 abstract report/ or letter/
48 or/45-47
49 44 not 48
50 25 and 49
51 (2008$ or 2009$).em.

**Appendix 4. PsycINFO**

1. exp endocrine sexual disorders/
2. Polycystic Ovar$.tw.
3. PCO$.tw.
4. (stein-leventhal or leventhal).tw.
5. (ovar$ adj1 sclerocystic).tw.
6. (ovar$ adj1 degeneration).tw.
7. exp statins/
8. HMG coenzyme reductase.tw.
9. hydroxymethylglutaryl-coa reductase inhibitor$.tw.
10. statin$.tw.
11. (Atorvastatin or Simvastatin).tw.
12. (Rosuvastatin or Lovastatin).tw.
13. (Mevastatin or Pravastatin).tw.
14. mevinolin.tw.
15. HMG-coA reductase$.tw.
16. HMG coenzyme A reductase.tw.
17. Zocor.tw.
18. meglutol.tw.
19. Hydroxymethylglutaryl CoA Reductase.tw.
20. Hydroxy 3 methylglutaryl CoA Reductase.tw.
21. CoA Reductase, 3-Hydroxy-3-methylglutaryl.tw.
22. Reductase, 3-Hydroxy-3-methylglutaryl CoA.tw.
23. or/1-6
24. or/7-22
25. 23 and 24
